# Integration of an Artificial Intelligence–Based Autism Diagnostic Device into the ECHO Autism Primary Care Workflow: Prospective Observational Study

**DOI:** 10.2196/80733

**Published:** 2025-10-21

**Authors:** Kristin Sohl, Erik Linstead, Kelianne Heinz, Elia Eiroa Lledo, Alicia Brewer Curran, Melissa Mahurin, Valeria Nanclares-Nogués, Carmela Salomon, Minda Seal, Sharief Taraman

**Affiliations:** 1 ECHO Autism Communities, University of Missouri School of Medicine, Columbia, MO, United States; 2 Fowler School of Engineering, Chapman University, Orange, CA, United States; 3 Cognoa Inc, 5000 Campus Drive, Newport Beach, CA, 92663, United States, 1 6507852624; 4 Pediatric Neurology and Clinical Informatics, Children’s Health of Orange County, Orange, CA, United States; 5 School of Medicine, University of California Irvine, Irvine, CA, United States

**Keywords:** autism spectrum disorder, diagnosis, computer-assisted, primary health care, artificial intelligence, machine learning, developmental disabilities, early diagnosis, real-world evidence, pediatric primary care

## Abstract

**Background:**

Pediatric specialist shortages and rapidly rising autism prevalence rates have compelled primary care clinicians to consider playing a greater role in the autism diagnostic process. The ECHO Autism: Early Diagnosis Program (EDx) prepares clinicians to screen, evaluate, differentiate, diagnose, and provide longitudinal care for children with autism in primary care settings. Canvas Dx is a prescription-only Software as a Medical Device designed to support clinical diagnosis or rule out of autism, including in primary care settings. It is authorized by the Food and Drug Administration for use, in conjunction with clinical judgment, in 18‐ to 72-month-olds with indicators of developmental delay.

**Objective:**

This study aims to assess the feasibility and impact of integrating the device into the ECHO Autism: EDx workflow. Time from the first clinical question of developmental delay to autism diagnosis is the primary endpoint. Secondary endpoints explore clinician and caregiver experience of device use.

**Methods:**

Children aged 18 to 72 months with concern for developmental delay indicated by either a caregiver or health professionals were eligible to participate in this prospective observational study. Experienced ECHO Autism: EDx clinicians were recruited to evaluate the inclusion of the device as part of their diagnostic evaluations. Outcome data were collected via a combination of electronic questionnaires, standard clinical care record reviews, and analysis of device outputs. Institutional review board approval was provided by the University of Missouri-Columbia (project number 2075722).

**Results:**

Eighty children and 7 clinicians completed the study. On average, time from clinical concern at study enrollment to final autism diagnosis was 39.22 days, compared to 180‐ to 264-day waits at adjacent specialist referral centers. The vast majority (93%, 50/54) of caregivers reported being satisfied with the ECHO Autism: EDx plus device evaluation their child received and endorsed that they would recommend it to others and that they felt comfortable using the device. The device produced determinate autism predictions or rule-outs for 52.5% of participants, and in all cases, these were consistent with the final clinical determination. Participating clinicians reported that device use was feasible and reduced several challenges associated with their previous diagnostic process; however, they noted it did not obviate the need for additional structured observation in every case.

**Conclusions:**

The ECHO Autism: EDx plus device workflow offers considerable time savings compared to specialty center referral and was strongly endorsed by caregiver participants. Embedding the device into the ECHO Autism: EDx workflow was feasible and helped streamline several workflow efficiencies. Clinicians still utilized their training and application and interpretation of Diagnostic and Statistical Manual of Mental Disorders, Fifth Edition, criteria when formulating the diagnosis for indeterminate cases.

## Introduction

### Background

Rates of children diagnosed with autism continue to rise dramatically, with one in 31 eight-year-olds now receiving a diagnosis in the United States [1]. While earlier identification and intervention during the critical neurodevelopmental window can maximize outcomes [2-4], many children experience critical delays to diagnosis [5]. There is a multiyear lag, for example, between the 18‐ to 24-month period where diagnosis is possible [6] and the average US age of diagnosis of nearly 5 years [7]. Delayed diagnoses represent missed opportunities for impactful early intervention, with new evidence emerging that even small delays in treatment initiation (18 vs 27 mo) can reduce its efficacy [8].

In the United States, autism evaluations typically occur in specialist centers, with less than 1% being made in primary care [9,10]. The inadequate number of specialists available to assess a rapidly growing pool of children with questions about developmental delay has created multiyear waitlists for evaluations [11,12]. To help address this crisis, calls to recruit more primary care physicians and practitioners to participate in the diagnostic process are growing [13-15].

While multiple studies show benefits to this approach, unanswered questions remain about the best way to integrate autism evaluations into the primary care workflow [16-20]. Many traditional diagnostic tools, for example, are time-intensive or difficult to administer [21] and thus represent barriers to autism evaluation in primary care. Two of the most commonly used tests in autism diagnosis are the Autism Diagnostic Interview-Revised and the Autism Diagnostic Observation Schedule [21]. The Autism Diagnostic Interview-Revised is a structured parent interview that typically takes 2‐2.5 hours to administer and score. The Autism Diagnostic Observation Schedule is an observational assessment that must be conducted in-person and can take 60‐90 minutes or longer to complete. Both instruments require formal in-person training followed by ongoing practice to maintain scoring reliability. Best results are often achieved by administering both tools to the same child [21], resulting in a time commitment that is unfeasible in the context of a primary care workflow.

### Canvas Dx

Canvas Dx, a Food and Drug Administration–authorized, artificial intelligence (AI)-based Software as a Medical Device, was developed to support streamlined autism evaluations in 18‐ to 72-month-olds with developmental delay concern [22]. The underlying machine learning algorithm was trained on data from thousands of children with diverse demographic backgrounds and behavioral health presentations with the goal of supporting accurate, equitable, and rapid identification or rule out of autism in primary care [23]. Canvas Dx does not require specialist training to administer and has been clinically validated for both remote and in-person administration [24].

The device’s gradient-boosted decision trees algorithm [23,25] integrates data from a brief caregiver questionnaire, a video analyst questionnaire, and a clinical questionnaire that can be administered either remotely or in person, while the clinician observes and interacts with the child. Where sufficient information is provided to make a highly accurate autism prediction or rule out, the device produces a *Positive for autism* or *Negative for autism* output. When a highly predictive determination cannot be made, the device abstains from providing a result (*Indeterminate* output).

The ECHO Autism Primary Care Early Diagnosis (EDx) Model [26,27] is a multifaceted professional development model including in-person and virtual components to support primary care clinicians regarding best practice autism evaluation and longitudinal care. The EDx model involves training and mentoring community-based clinicians to conduct diagnostic assessments for autism, consistent with best practice standards. ECHO Autism: EDx is the first of its kind model designed to professionally develop community-based primary care clinicians to assess and appropriately diagnose young children with a question of autism. However, it requires significant resources of time and professional learning to achieve competency in serving this population well. Therefore, ECHO Autism Communities and Cognoa engaged in this study to evaluate the feasibility of using Canvas Dx in lieu of the model’s usual observational tool, the Screening Tool for Autism in Toddlers and Young Children (STAT) [28], to support primary care clinician diagnostic determination.

The primary study objective was to assess the time from initial clinician concern to diagnosis when using the device as part of the ECHO Autism: EDx diagnostic pathway. The secondary objective was to explore clinician and caregiver experience of the feasibility and impact of device use as part of the diagnostic journey.

## Methods

### Study Protocol Overview

The study protocol for this prospective observational feasibility study is described in full in JMIR research protocols [29], with key methodological considerations summarized further. Eligible participants were enrolled consecutively to minimize selection bias, with data collection occurring between April 2022 and November 2023.

### Ethical Considerations

The study received institutional review board approval from the University of Missouri-Columbia (project number 2075722), and the protocol was registered with ClinicalTrials.gov prior to study initiation (protocol identifier: NCT05223374). Written informed consent was obtained from all participants, and the participants had an ability to opt out of the study at any time. Device costs were covered by the study sponsor Cognoa, but no other monetary compensation was provided to the participants. Data have been anonymized to protect participant confidentiality, and no identifiable images of participants are included in the manuscript or supplementary material.

### Blinding

Cognoa leadership and ECHO Autism leadership were blinded to the study dataset and had no access to the study end points until after the first data analysis was completed by an independent third party. This was to ensure minimal introduction of influence by either organization involved in the study.

### Inclusion Criteria

#### ECHO Autism Clinicians

ECHO autism clinicians (EACs) eligible for study participation were practicing primary care physicians who had previously received training in the ECHO Autism: EDx model and had met diagnostic agreement definitions to independently evaluate and diagnose autism [30].

#### Patients

As part of routine practice, participating clinicians received referrals to evaluate pediatric patients. Staff at participating clinics screened referred children during the study period to assess if they potentially met the criteria for study inclusion. If the criteria appeared to be met, clinic personnel informed families about the study. Interested families were subsequently referred to the University of Missouri ECHO Autism Communities Research Team, who were responsible for reviewing the study in detail and obtaining informed consent from the caregiver prior to participation.

Children were eligible for enrollment if, at the time of screening, they were between 18 and 72 months of age and a caregiver, health care provider, or community professional had expressed concern about possible developmental delay. Eligible participants were required to come from families proficient in English or Spanish, as determined by the assessing EAC, and a parent, guardian, or legally authorized representative needed to be capable of reading and signing informed consent. Families also had to have access to a smartphone operating on a recent version of iOS or Android, be willing to provide short home video recordings of the child for device input, and meet all device labeling requirements [31].

Children were excluded post-screening if they had a previous autism diagnosis provided by a health care professional or if they had a medical, developmental, or behavioral condition that, in the opinion of the EAC, could interfere with study assessments. Any potential participant who had previously been involved in any study or survey related to the Canvas Dx device was also excluded. Additionally, patients were excluded if they had conditions listed as exclusions for reliable use by the device manufacturer. These conditions included suspected auditory or visual hallucinations or a prior diagnosis of childhood-onset schizophrenia; known deafness or blindness; physical impairments affecting hand use; major dysmorphic features or prenatal exposure to teratogens (eg, fetal alcohol syndrome); history or diagnosis of genetic conditions such as Rett syndrome or Fragile X; microcephaly; history of epilepsy or seizures; history of neglect; and brain defects, injuries, or insults requiring interventions such as surgery or chronic medication.

### Data Collection Instruments

The device inputs consist of the following:

1. A *caregiver questionnaire* consisting of 18- or 21-item (age-dependent) multiple questions that capture the caregiver’s observations of the child’s development and behaviors. Caregivers independently completed the questionnaire within a mobile app and were blinded to other device inputs. The caregiver questionnaire is designed to be used in combination with other device inputs rather than as a stand-alone assessment.
2. A video analyst questionnaire answered based on analysis of two videos the caregiver uploads via the mobile app. The 28- or 33-item questionnaire (age-dependent) is completed by trained video analysts within the analyst portal. Video analysts are required to have at least a master's degree, 5 or more years of experience working with children with autism, and must undergo training and yearly recertifications. Each video is independently scored, with analysts documenting behaviors across relevant domains, including verbal and nonverbal communication, social interaction, sensory responses, repetitive movements, use of objects, and speech. Video analysts are blinded to the caregiver questionnaire responses and clinician questionnaire responses and must answer based solely on their observations of the videos.
3. A *clinical questionnaire* consisting of 13 to 15 (age-dependent) multiple choice questions is answered by the EAC during an observational session with the caregiver and child. The clinician is blinded to the results of the video analyst questionnaire and must answer based on their observations of the child’s behavioral and developmental characteristics and knowledge of the child’s history. The EAC inputs questionnaire responses via a web portal. The clinical questionnaire is one of the required device inputs and is not a stand-alone measurement tool.

The device output is accessed via the clinical web portal. It consists of a diagnostic determination (Positive for autism, Negative for autism, or Indeterminate); all questions and responses from Inputs 1 and 3; and the caregiver’s submitted videos.

Measure of Processes of Care: This is a validated tool adapted for this study to measure caregivers’ perceptions of the family-centeredness of the care provided.

ECHO Autism Caregiver Presurvey: This collects baseline demographic information and captures caregivers’ initial impressions of the EAC prior to the diagnostic process.

ECHO Autism Caregiver Postdiagnostic Survey: This assesses caregiver satisfaction with the diagnostic services received.

EAC Pre-Project Survey: This evaluates clinicians’ self-efficacy, perceived barriers, and satisfaction with the current ECHO Autism diagnostic process. The current ECHO autism diagnostic workflow consists of the following: (a) completion of a 2-day in-person training on the STAT. This is a direct structured behavioral observation that takes approximately 20 minutes to administer in the physician’s office once training is complete; (b) achievement of administration and coding reliability on the STAT instrument to a certified STAT trainer; (c) participation in twice monthly, 90-minute iterative case-based learning through ECHO Autism: Primary care sessions where additional skills required as part of best practice autism evaluations such as diagnostic interviewing, differential diagnosis, and conceptualization of development and behavior in young children are acquired.

EAC Post-Project Survey: This assesses changes in clinician self-efficacy, perceived barriers, and satisfaction following integration of the device into the diagnostic workflow.

ECHO Autism 3-Month Follow-Up Questionnaires: This was designed to capture data needed to address secondary and exploratory study objectives.

ECHO Autism Interim Visit Form: This was used to document any participant visits occurring between the initial concern and the 3-month follow-up.

### Statistical Analysis

Descriptive statistics were used to summarize all collected survey data from caregiver and clinician participants. Continuous variables are reported as n, mean, standard deviation, median, minimum, and maximum values, while categorical variables are presented as counts and percentages where relevant. All available data, including observed and missing values, were tabulated.

Time from initial clinical concern at enrollment to diagnosis by the EAC was calculated in days and compared with the projected wait time if participants had been referred to adjacent specialist centers at first concern instead of following the primary care diagnosis pathway. Both EACs and adjacent specialist centers assess children with concern for developmental delay across the complexity spectrum and labeling age range. To generate comparison estimates for specialty center wait times, we obtained publicly reported median wait times to first appointment from the Missouri Autism Specialty Centers [32]. The Missouri Autism Centers aggregate and track median wait time data from multiple autism specialty centers across Missouri. Data are reported annually by fiscal year and stratified into two age cohorts: children under 60 months and children 60 months and older. Because the current study period spanned two fiscal years (2022‐2023), we calculated the mean of the 2 years’ reported median wait times to derive an overall estimate for each age cohort. For children under 60 months, the reported median wait times were 143 days (FY2022) and 217 days (FY2023), yielding an average of 180 days. For children 60 months and older, the reported medians were 221 days (FY2022) and 306 days (FY2023), yielding an average of 264 days. These averaged values were used as the comparison benchmarks in subsequent analyses. Distance from the families’ home address to the primary care EAC appointment was collected as part of the caregiver surveys and compared with the distance families would have otherwise been required to travel to their nearest specialist center.

Device diagnostic determinations (*Positive for autism, Negative for autism*, or *Indeterminate*) were compared with the reference standard diagnosis from the EAC to calculate positive and negative predictive values, as well as sensitivity and specificity in the determinate group. Device performance was also analyzed across race or ethnicity, biological sex, and age to determine if performance metrics varied by demographic. Device-generated timestamps were used to assess the time burden associated with input completion.

### Study Flow

**Figure 1.**
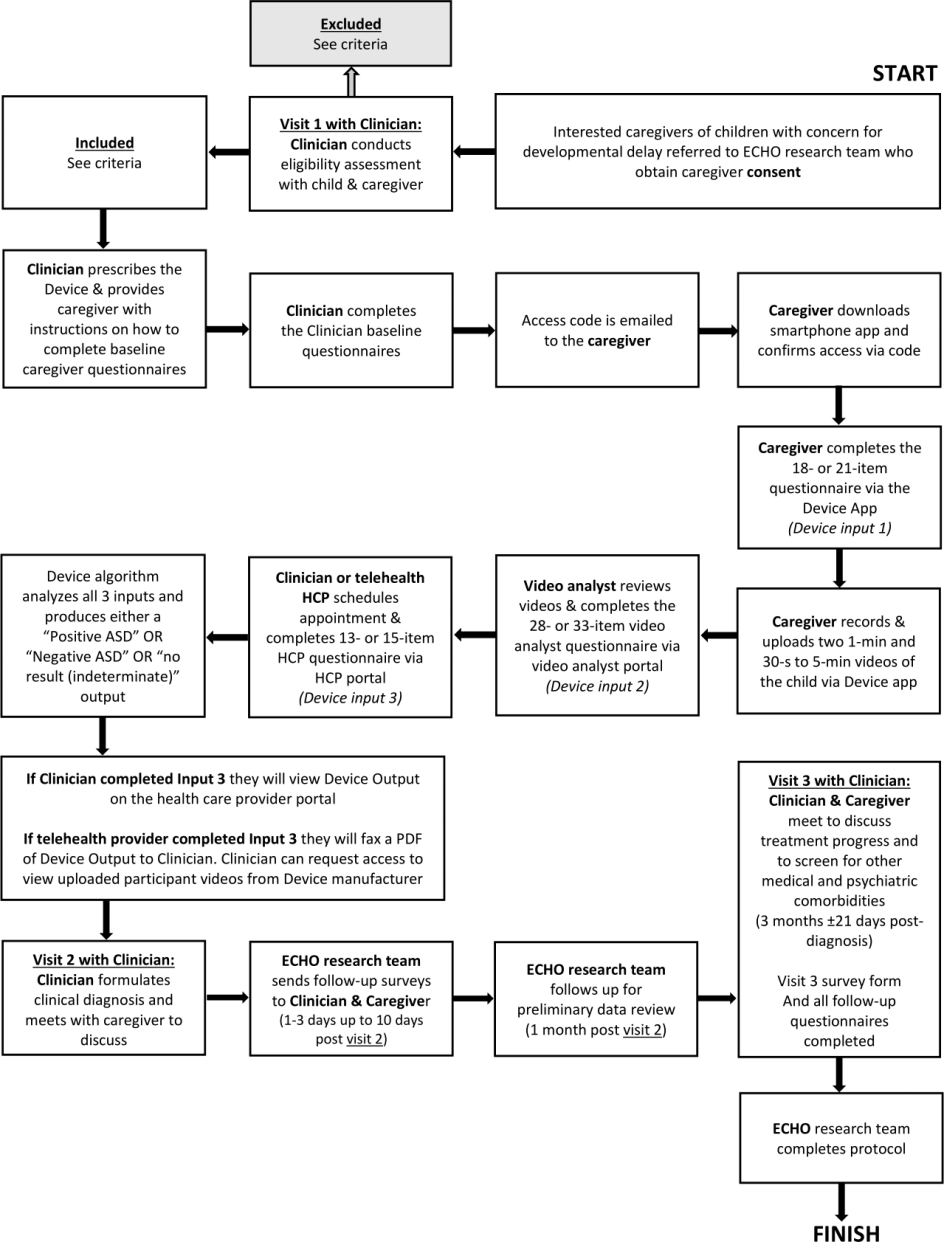
Study flow overview for prospective observational study of Canvas Dx integration into primary care workflow to support diagnosis or rule out of autism: Caregivers of children with developmental concerns (18-72 mo) are referred and consented by study team. If deemed eligible during Visit 1 (observation and DSM-5 interview), the clinician prescribes the device. The device captures three inputs: (a) caregiver questionnaire+home videos (Input 1), (b) remote video-analyst scoring (Input 2), and (c) clinician questionnaire (Input 3)—to generate a positive/negative/indeterminate output. During Visit 2, the clinician makes a diagnostic determination based on device results and clinical impression. If positive for autism, Visit 3 is scheduled to review supports or services. DSM-5: Diagnostic and Statistical Manual of Mental Disorders, Fifth Edition.

Figure 1 summarizes the full study flow.

#### Prior to Visit 1

A clinical appointment is scheduled for children with a question of autism. Clinic staff share study information with eligible families, and those who expressed interest are referred to the research team, where informed consent is obtained.

#### Visit 1: Unstructured Observation and Comprehensive History

Participating ECHO Autism: EDx clinician observes the participant and conducts a comprehensive medical and developmental history along with Diagnostic and Statistical Manual of Mental Disorders, Fifth Edition (DSM-5)–based autism-focused behavioral interview. Visit one lasts 45 minutes, on average.

#### Device Completion

The child’s caregiver downloads the device mobile application to their smartphone and completes the Caregiver Questionnaire and video recording tasks. The uploaded videos are remotely reviewed and scored by trained video analysts via the video analyst portal. The clinician completes the clinical questionnaire on a web portal. The device algorithm analyzes all three inputs and produces either a Positive for autism, Negative for autism, or Indeterminate output.

#### Visit 2: Diagnostic Results

The clinician makes an autism diagnosis or rules it out after synthesizing the clinical information and the device output. For children with a confirmed positive result, the EAC will make the diagnosis in primary care and recommend treatments as appropriate during this visit. For children with a negative result, the EAC will rule out autism. If warranted, they may assess for other possible causes of developmental delay and recommend treatments accordingly. For children with an indeterminate result, the EAC will rely on their observations, comprehensive DSM-5 history, and clinical expertise to arrive at an autism status determination. The clinician reviews the diagnostic determination with the caregiver. If the child receives an autism diagnosis, the clinician will schedule a 3-month follow-up visit. The research team sends electronic post-diagnostic questionnaires to the family and the clinician.

#### Visit 3: Supports and Services Progress Meeting

The patient and their caregiver will follow up with the clinician 3 months (±21 d) after they receive an autism diagnosis to discuss progress associated with obtaining and receiving supports and services. Additionally, the clinician will continue to screen and monitor for common medical and psychiatric co-occurring conditions. The research team will send electronic post-diagnostic questionnaires to the caregiver and to the clinician to be completed 1‐3 (up to 10) days after this follow-up visit.

### Protocol Deviations

The protocol anticipated a data-collection period of approximately 12 months and a target of about 100 participants completing the full protocol. These were planning projections and not required minimums or formal stopping rules. In practice, 109 participants were enrolled and 80 completed all study procedures. Because the study had already extended beyond the anticipated 12-month window, we concluded data collection at 80 completers due to operational constraints. This deviation pertains only to study duration and completer count. No changes were made to eligibility criteria, data collection procedures, or the prespecified analysis plan.

## Results

### Participants

**Figure 2.**
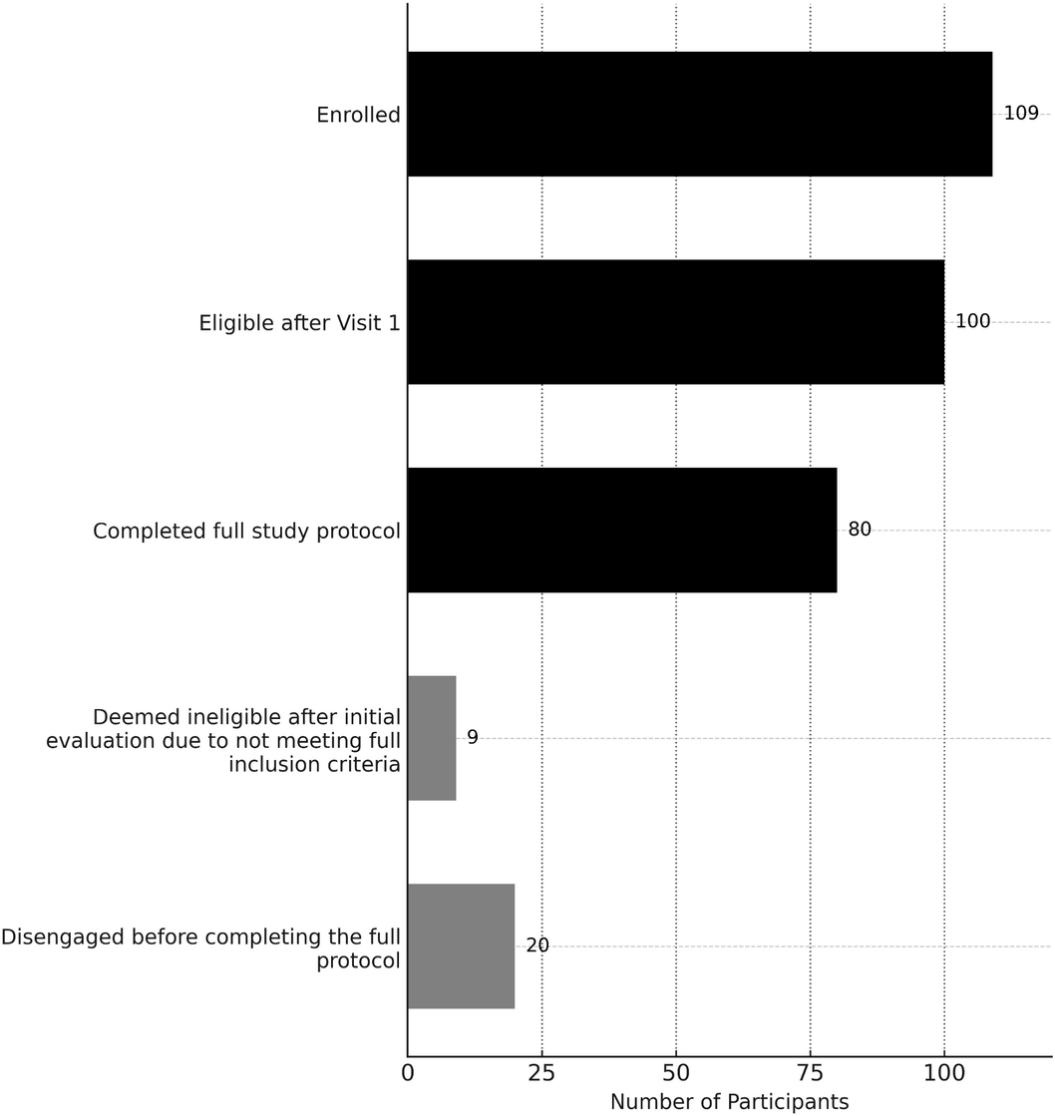
Participant recruitment and retention funnel: A total of 109 patients aged 18-72 mo with concern for developmental delay initially enrolled. One hundred of these were deemed eligible to participate in the full study after visit 1. Eighty of these eligible patients completed all device inputs and held a diagnostic results meeting with their clinician (80% of total eligible enrolled participants).

Figure 2 summarizes the recruitment and retention funnel for the study.

### Patient Demographics

The overall patient population had an autism prevalence of 60% (48/80) per clinician evaluation and was 30% (24/80) female. The age of participants ranged from 18.29 to 71.28 months at first appointment, with a mean age of 42.25 (SD 14.41) months and median age of 39.91 months. There were no gender or age-related differences between study completers and noncompleters. Just over half (61.76%, 63/102) of participants were primarily insured by Medicaid, while 38.24% (39/102) of participants were privately insured (Table 1).

**Table 1.** Patients’ primary insurance, shown for the full population and also stratified by study completion status.

Population	Medicaid primary insurance, n (%)	Private primary insurance, n (%)
All patients (N=102)	63 (61.76)	39 (38.24)
Completers (N=80)	49 (61.25)	31 (38.75)
Noncompleters (N=22)	14 (63.64)	8 (36.36)

### Caregiver Demographics

Demographic data were collected from the caregivers of most enrolled patient participants (n=104; Table 2). Most caregivers reported being White or Caucasian (85.58%, 89/104). This likely reflects the demographic makeup of Missouri where approximately 80% of the population identify as “White Non-Hispanic” [33]. Most caregivers reported a highest education level attained of high school graduate or GED or some college but no degree (28.85% [30/104] and 28.85% [30/104], respectively). The majority of caregivers reported a household income between US $25,000 and 49,999 or US $0‐24,999 (38.46% [40/104] and 26.92% [28/104], respectively). The age of caregivers ranged from 20 to 49 years, with a mean age of 31.86 (SD 6.36) years and median age of 31. The demographic distribution of the caregivers of participants who completed the trial was similar to that of the caregivers of all enrolled participants.

**Table 2.** Caregiver race, educational attainment, and household income demographics, shown for the full population and stratified by study completion status.

Caregiver’s demographics	Percent of enrolled participants^a^, n (%)	Percent of trial complete participants^b^, n (%)
Race		
American Indian or Alaska Native	3 (2.88)	3 (3.75)
Asian	2 (1.92)	2 (2.5)
Black or African American	14 (13.46)	10 (12.5)
Native Hawaiian or Other Pacific Islander	2 (1.92)	1 (1.25)
White or Caucasian	90 (86.54)	70 (87.50)
Educational attainment		
Less than high school graduate	6 (5.77)	5 (6.25)
High school graduate or GED^c^	30 (28.85)	23 (28.75)
Some college, no degree	30 (28.85)	21 (26.25)
Bachelor’s degree	31 (12.5)	12 (15)
Trade or technical school graduate	12 (11.54)	9 (11.25)
Post graduate degree or higher	13 (12.5)	10 (12.5)
Household income (USD)		
0‐24,999	28 (26.92)	20 (25)
25,000‐49,999	40 (38.46)	31 (38.75)
50,000‐74,999	13 (12.5)	11 (13.75)
75,000‐99,999	9 (8.65)	7 (8.75)
100,000 and over	14 (13.46)	11 (13.75)

a N=104.

b N=80.

c GED: General Educational Development ( high school equivalency).

#### ECHO Autism Clinician Demographics

Seven clinicians participated in this study. Each participating primary care physician completed rigorous mentoring in using the ECHO Autism: EDx model. The majority of participating clinicians (71.43%, 5/7) were female, and 100% were White or Caucasian (not Hispanic or Latino). The age of clinicians ranged from 37 to 72 years, with a mean age of 48.43 (SD 13.51) years and median age of 42 years.

### Time From Initial Clinical Question to Autism Diagnosis

#### ECHO Autism: EDx Workflow With Embedded Device

The total average time from clinical question at study enrollment to final autism diagnosis was 39.22 (SD 57.08) days. The most frequent time elapsed between enrollment and diagnosis was 14 days, and the median time was 13.98 days.

Clinicians spent an average of 6 minutes and 20 seconds (SD 4 min and 36 s) completing the clinical device questionnaire, compared to the typical 20 minutes they previously spent completing the STAT instrument. Caregivers spent an average of 6 minutes and 44 seconds (SD 5 min and 41 s) completing the caregiver device questionnaire. The time spent by caregivers on their questionnaire ranged from a minimum time of 1 minute and 43 seconds to a maximum time of 37 minutes and 40 seconds and a median time of 4 minutes and 44 seconds.

#### Comparison to Adjacent Specialty Referral Center Wait Times

In the period the study was conducted, adjacent specialty referral centers reported wait times of 180 to 264 days. If participants with a question of delayed development had been referred onto these waitlists after visit one, rather than being evaluated in primary care, their diagnoses would have been delayed by 141‐225 days.

### Device Performance Versus Process as Usual

Participating clinicians provided a definitive autism diagnosis or rule out to all study completers in the primary care setting. The device provided determinate results (ie, positive for autism or negative for autism) for 52.50% (42/80) of patients. Determinate device outputs agreed with the clinician diagnosis in 100% of cases, giving a positive predictive value, negative predictive value, sensitivity, and specificity of 100% (Table 3). The device did not produce any false positives or false negatives. Most indeterminate patients received a negative autism diagnosis from their clinician (78.95%, 30/38). There were no significant differences in the rate at which the device output a determinate versus an indeterminate result based on participants’ age (under 48 mo vs over 48 mo; insignificant, P=.36), sex (male vs female; insignificant, P=.81), race or ethnicity (insignificant, P=.78) or socioeconomic status (income brackets 0‐50k, 50k-100k, 100k+; insignificant, P=.87*).*

**Table 3.** Device output (positive or negative or indeterminate for autism) versus final clinical diagnosis (positive or negative for autism).

	Clinician diagnosis, n (%)	
Device output	Positive autism (n=48)	Negative autism (n=40)
Positive autism (N=40)	40 (100)	0 (0)
Indeterminate (N=38)	8 (21.1)	30 (79)
Negative autism (N=2)	0 (0)	2 (100)

The STAT, in contrast, generates a low or high-risk score for each child (binary classifier); however, without a built-in ability to abstain in cases of high uncertainty, its overall predictive performance for identifying or ruling out autism is significantly lower than that of the device. .

### Caregiver Experience

#### Satisfaction and Likeliness to Recommend

Caregivers reported positive experiences using the device and throughout the clinician evaluation. The vast majority (92.59%, 50/54) of caregivers reported being satisfied (agreed or strongly agreed) with the evaluation their child received from their clinician and endorsed that they would recommend this type of evaluation process to others (92.86%, 65/70). Additionally, 91.78% (67/73) stated they felt comfortable (agreed or strongly agreed) using the device as part of their child’s evaluation. The majority of caregivers agreed or strongly agreed that the device app worked without difficulties (81.94%, 59/72). Few users reported issues with filming instructions (11.11%, 8/72), required assistance to use the device (7.14%, 5/70), or had issues with internet connectivity while using the device (8.58%, 6/70)

#### Impact of Assessment Location on Distance Traveled

Specialty centers required traveling an average of 96.82 (SD 78.38) miles from participants’ residences. However, caregivers reported traveling only 51.98 (SD 75.69) miles, on average, to the in-person appointment with their primary care clinician. This is an average of 44.84 miles saved compared to the distance they would have traveled if assessed at the nearest specialty center. The vast majority (92.59%) of caregivers agreed that it was helpful to have their child evaluated in the community by an EAC instead of traveling to a specialist center.

### Clinician Experience

Survey data were collected from all seven clinician participants at the start of the study and at the end of the study and in relation to each patient who received an evaluation using the device. Clinicians agreed with the output provided by the device for 100% of determinate cases (Table 4). Most (71.43%, 5/7) clinicians agreed that the device worked well within their clinical practice flow, and more than half (57.14%, 4/7) felt that the device added information to their diagnostic impression.

**Table 4.** Level of clinician agreement with determinate device output.

Clinician agreement with device output	Determinate output patients (N=42), n (%)
Strongly agree	29 (69.05)
Agree	13 (30.95)
Neutral or no opinion	0 (0)
Disagree	0 (0)
Strongly disagree	0 (0)

Clinicians reported that barriers associated with using their previous process (ie, STAT) “process as usual” were reduced when administering the device (Table 5). Time, for example, was identified as a barrier by 71.43% (5/7) of clinicians for the process as usual compared to only 28.57% (2/7) for the device. Similarly, child cooperation was reported as a barrier by 42.86% (3/7) of clinicians for ECHO Autism: EDx process as usual but was decreased to 28.57% (2/7) with the use of the device. Family resistance, reported by 14.29% (1/7) of clinicians as a barrier for process as usual, was not reported as a barrier when using the device. On the other hand, 85.71% (6/7) of clinicians reported technology as a potential barrier with device use, likely because families are required to download an app and independently complete in-app tasks, whereas the STAT was a pen and paper test administered by the clinician.

Additional barriers unique to the device included insufficient clinical information from caregiver videos and limited usefulness of the output generated by the device in cases when an indeterminate result was generated (Table 5). Just over half of clinicians (57.14%, 4/7) reported being unlikely to uniformly recommend the device in lieu of additional direct observation for these cases. While just under half (42.86%, 3/7) of the participating physicians agreed that the device saved them time in the overall diagnostic process, 28.57% (2/7) were uncertain and 28.57% (2/7) disagreed.

**Table 5.** Clinician-reported barriers to diagnosis using ECHO autism: EDx process as usual versus the device (N=7).

Barrier	EDx process as usual, n (%)	EDx plus the device, n (%)
Time	5 (71.43)	2 (28.57)
Patient uncooperative	3 (42.86)	2 (28.57)
Resistance from family	1 (14.29)	0 (0)
Technology	0 (0)	6 (85.71)

## Discussion

### Main Findings

This collaborative observational study was the first to evaluate the use of a novel AI-based autism diagnostic device as part of the ECHO Autism: EDx workflow. Compared to specialty center referral, these primary care–based autism evaluations reduced the time from first clinical concern to diagnosis by approximately 5‐9 months. Families, on average, traveled 45 miles less to each appointment than they would have if assessed at their nearest specialty center. Participating caregivers reported strong levels of satisfaction with both the device itself and the ECHO Autism diagnostic process more broadly, highlighting that evaluation in a primary care setting was acceptable to families. High levels of caregiver satisfaction with primary care autism evaluations [34] reduced travel burden [35] and significantly shortened time from first concern to diagnosis [36] are consistent with previously published topical study findings.

While most clinicians endorsed that device use was feasible and provided workflow efficiencies compared to their previous process, clinician participants noted device use did not always obviate the need for additional observations. While there was 100% alignment between determinate device predictions and the final clinical diagnosis, in the roughly half of cases where the device abstained from making an autism prediction or rule out, clinicians were required to rely more heavily on their observations, comprehensive DSM-5 history, and clinical expertise to arrive at a diagnostic determination. This emphasizes the need for clinicians seeking to include autism diagnosis in their practices to obtain and maintain the clinical expertise necessary to interpret varied result outputs in their diagnostic determination process.

Technological barriers to device use noted by a minority of caregiver participants included issues with video uploads, video recording directions, and unreliable internet access. Since the time of study completion, the device manufacturers have implemented a number of updates to improve video uploading functionality and in-app recording directions. Future testing to determine the impact of these updates on user experience is recommended.

### Limitations

The version of the device assessed in this study included only a positive, negative, or indeterminate output but did not include an accompanying detailed report explaining the result. Since the time of study completion, supporting software has been developed that increases the transparency of the device’s AI, mapping collected data to DSM-5 criteria relevant to autism diagnosis and auto-generating a detailed report to reduce clinical documentation burden [25]. Decision threshold updates have also been made to reduce the number of indeterminate outputs that are returned [23]. Future clinician users’ perceptions of device utility and efficiencies (particularly in indeterminate cases) may differ from those reported by study participants, given this significant update in functionality.

Additionally, we note that the clinician sample size (7) was small, and their demographics were not representative of the broader US population, with all being White or Caucasian. Clinician study participation was impacted by EACs being in primarily nonacademic, rural communities with less infrastructure and familiarity with participating in research. Currently, the device is only available in English and Spanish, so findings reported here may not generalize to other non-English or non-Spanish speaking populations. While no significant differences in device abstention rates were noted across age, sex, socioeconomic status, or race and ethnicity, the authors recommend that larger studies be conducted to confirm these findings in more racially diverse samples.

### Conclusion

The ECHO Autism: EDx plus device workflow was feasible and reduced barriers to diagnosis for both caregiver and clinician users. Compared to referral to adjacent specialist waitlists, use of the ECHO Autism: EDx plus device workflow resulted in a reduced travel burden for families and significantly shortened patient delays from first clinical question to diagnosis and treatment initiation.

These findings may be of significant interest to families with children stuck on long wait lists for autism evaluations, as well as policymakers, clinicians, and payors seeking to find solutions to the current waitlist crisis. Since the Centers for Disease Control and Prevention began tracking rates of autism among 8-year-olds in 2000, prevalence rates have increased by over 300% [37,38]. Meanwhile, the specialist workforce, typically responsible for conducting autism evaluations, has not kept pace with this increase. Currently, there is only an estimated one developmental behavioral pediatrician per 100,000 children [11] and 14 Child and Adolescent Psychiatrists per 100,000 children [39]. This prospective observational study demonstrated that innovative professional development models like ECHO Autism, combined with emerging diagnostic technologies like Canvas Dx, can help equip the much larger primary care workforce to feasibly play a greater role in diagnosing and managing autism without automatic specialist referral. Strategies to further expand the pool of qualified primary care clinicians able to participate in the diagnostic process, and funding to equip them with evidence-based diagnostic supports, should be pursued to unblock specialist waitlists and support timely treatment initiation.

## Abbreviations

AI: artificial intelligence
EAC: ECHO autism clinician
EDx: Early Diagnosis Program
STAT: Screening Tool for Autism in Toddlers and Young Children
